# Back-to-Germline (B2G) Procedure for Antibody Devolution

**DOI:** 10.3390/antib8030045

**Published:** 2019-08-26

**Authors:** Anja Schrade, Alexander Bujotzek, Christian Spick, Martina Wagner, Johannes Goerl, Xenia Wezler, Guy Georges, Roland E. Kontermann, Ulrich Brinkmann

**Affiliations:** 1 Roche Pharma Research and Early Development (pRED), Large Molecule Research (LMR), Roche Innovation Center Munich, 82377 Penzberg, Germany; 2 Institute of Cell Biology & Immunology, Stuttgart University, 70569 Stuttgart, Germany

**Keywords:** protein engineering, antibody, maturation, affinity, structure, antigen binding

## Abstract

Bispecific antibodies (bsAbs) with avidity-enhanced specificity can be used to address target cells with increased specificity, ideally binding efficiently to cells that express two cognate antigens, yet not to cells that express only one of those. Building blocks required to generate such bsAbs are binders that recognize the two antigens with high specificity yet with various (including very low monovalent) affinities. The herein described ‘back-to-germline’ (B2G) procedure defines such derivatives. It converts parent antibodies with high specificity to derivatives that retain specificity but modulate affinity. The approach defines mutations to be introduced into antibody complementarity-determining regions (CDRs) regions without requiring structures of antibody-antigen complexes. Instead, it reverses the B-cell maturation process that increases affinities, with preference on CDR residues with high antigen contact probability. Placing germline residues at those positions generates VH and VL domains and Fv-combinations thereof that retain specificities but are ‘de-matured’ to different degrees. De-maturation influences on-rates and off-rates, and can produce entities with extremely low affinity for which binding can only be detected in bivalent formats. A comparison with alanine replacement in CDRs (so far, the most frequently applied technology) indicates that B2G may be more reliable/predictable without introduction of stickiness or poly-reactivity. The applicability for generating sets of affinity-modulated monospecific variants is exemplarily shown for antibodies that bind CD138, Her2/neu, and EGFR.

## 1. Introduction

Combination therapy of two monoclonal antibodies (mAbs), including cetuximab (Erbitux) and trastuzumab (Herceptin) have shown promising results to treat solid tumors known to be dependent on the expression of tyrosine kinase receptors EGFR and HER2 [1]. However, the administration of two mAbs requires an individual regulatory review and approval. The next generation of antibody therapeutics, therefore, include bispecific antibodies (bsAbs) featuring the ability to bind two targets at a time [2,3,4,5]. There are various scenarios how these bsAbs can improve therapeutic activity. One scenario is the ability to target specific immune cells or proteins in addition to a therapeutic target and, thus, enhance effector function or antibody delivery to a specific organ, respectively [6,7,8]. Other ways to improve antibody efficacy through bispecificity are by blocking two different biological pathways or simply increasing the specificity toward tumor cells by dual targeting of two individual cell surface antigens [9].

If an antigen is not exclusively displayed on target cells, but, additionally, on non-pathogenic cells. One option is to generate bsAbs that address a second antigen. The combination of both targets should only be present at the desired target site [10,11]. However, although preferential, bispecificity itself does not necessarily come with increased specificity. If monovalent binding of the bsAb is sufficient for high affinity binding, cells that express only one particular antigen can be bound. To achieve increased specificity by bispecificity and to avoid off-target effects, avidity needs to be the driver [12]. In an ideal setting, monovalent binding affinity is not sufficient to retain the antibody on the cell surface and only if both arms can bind to their specific antigens, then the therapeutic bsAbs are retained. The desired binding properties are known as the Avidity Mediated Specificity Gain (AMSG) principle and AMSG binders are entities that recognize a target antigen with high specificity yet with very low monovalent affinity [13]. Current approaches to generate such binding entities include, in many cases, either de-novo generation of antibodies or introduction of mutations to decrease affinities of existing antibodies. De-novo generation of antibodies with very low (in ideal cases, non-detectable) affinity in a monovalent format proves to be difficult and incompatible with standard antibody generation technologies, which require significant binding for detection. To circumvent this problem and generate AMSG-driven binding entities, one approach is to take existing specific binders and introduce mutations into the binding region to reduce affinity. For example, one can determine their structure, model antigen interactions, and introduce disturbing mutations. As of today, alanine scanning mutagenesis is the method of choice to explore protein–protein interfacial residues that can be applied to a wide variety of protein complexes to understand the structural and energetic characteristics [10,14]. The disadvantage of these approaches is either that the crystal structure of the antibody-antigen complex needs to be known or determined first, that the introduction of mutations without the knowledge of structure might disturb antibody structure dramatically, or that the alanine mutations introduce poly-reactivity [15,16]. Computational calculations of alanine replacement of charged amino acids, including arginine, aspartic acid, glutamic acid, lysine, and histidine often results in disagreement with values obtained from laboratory experiments [17,18].

**Figure 1:**
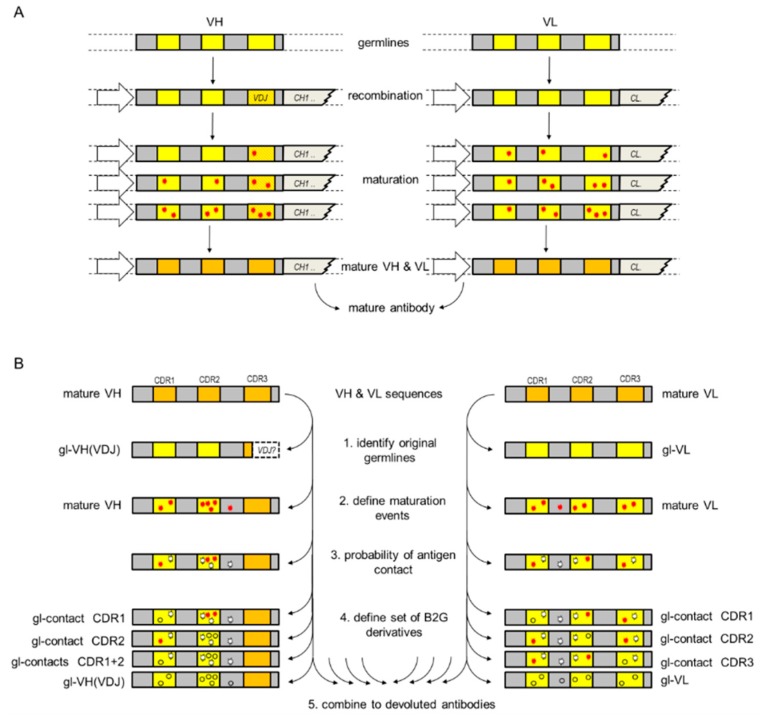
Antibody maturation (**A**) in B cells and (**B**) de-maturation by B2G.

Desired is a generalizable approach to generate a set of antibody derivatives with various affinities in the absence of poly-reactivity. In fact, such a set of antibody derivatives is generated by nature during the generation and maturation of antibodies in B-cells [19] (Figure 1A). During the first step, recombination generates a binder specific for a certain antigen, yet relatively low affinity. VDJ recombination generates initial H3 on a defined VH germline, combined with one defined VL. Multiple rounds of somatic hyper mutations (SHM) lead to an increased antibody affinity with selective pressure toward antigen binding in the germinal center. These maturation steps generate variations in antigen binding region, better binder selection, and lead to the evolution of the antibody. The affinity of the resulting mature antibody toward its antigen is increased by many orders of magnitude when compared to corresponding naïve B-cell receptors and its structure differs from germline-encoded counterparts [20]. Several studies, including crystallographic, spectroscopic, and kinetic investigations, indicate that mature antibodies are frequently more rigid when compared to germline encoded antibodies [21,22,23,24]. Whereas in germline antibodies, the loops of the complementarity-determining region (CDR) remain flexible and the mutated CDRs of mature antibodies are able to prearrange for binding [25].

Our approach, which aims to generate a set of antibody derivatives with reduced affinity but retaining specificity, is based on reversing B cell maturation. In consequence, the procedure described in this case generates antibody derivatives with antibody functionalities of different branches of the maturation tree, expected to have different affinities, yet retaining the specificity of the matured starting antibody (Figure 1B).

## 2. Materials and Methods

### 2.1. Antibodies

For the experiments described, protein sequences of monovalent IgG antibodies against hCD138, hHer2/neu, and hEGFR were identified in the literature (<CD138>, US9446146B2, <Her2/neu>, Trastuzumab, US6870034B2 [26]; <EGFR>, Cetuximab, US6217866B1). These sequences were used as master (parental IgG) to generate affinity variants. All antibodies were expressed transiently in non-adherent HEK-293 cells and purified using protein-A affinity and size-exclusion chromatography using an Äkta system (GE Healthcare) [27,28]. To verify antibody identity analytical size exclusion chromatography, SDS page analysis were performed (Figure S1) and electrospray ionization mass spectra were acquired on a maXis Q-TOF (Bruker Daltonics, Bremen, Germany) equipped with a TriVersa NanoMate (Advion, Ithaca, NY). Production yields are listed in Tables S1 and S4.

### 2.2. Surface Plasmon Resonance to Determine Antibody Kinetics

The kinetics of all antibody affinity variants (<hCD138>, <hHer2/neu>, and <hEGFR> to the extracellular matrix domain of the corresponding antigen was evaluated using a Biacore T200 instrument (GE Healthcare) [29]. Association and dissociation were determined for both in a monovalent (affinity-driven) and bivalent (avidity-driven) binding mode, as described below. To determine monovalent binding properties (1:1 interactions), antibodies and derivatives were captured via anti-hFc on chip surfaces followed by subsequent application of monovalent antigens, as detailed for individual antigens below. To determine bivalent binding properties (avidity), antigens (see details for individual antigens below) are coupled in high density to SPR chip surfaces, which is followed by subsequent application of bivalent antibodies and derivatives that need to be analyzed.

### 2.3. CD138 Affinity SPR

Solution of anti-CD138 antibody affinity variants 30 nM in HBS-P+ (GE Healthcare) were captured with anti-hFc antibody (GE Healthcare BR-1008-39) on a CM5 sensor chip for 60 s (capture Level ~ 900 RU). Thereafter, the interaction with hCD138 (R&D-S. 2780-TS) was analyzed in a dilution series of 66 nM to 600 nM using a 150-s association time and a 600-s dissociation time at a flow rate of 30 µL/min. All Biacore T200 experiments were carried out in HBS-P+ (GE Healthcare) pH 7.4 running buffer and at 25 °C. Binding curves were evaluated using T200 evaluation software. For the calculation of binding properties, a 1:1 Langmuir binding model was used. All kinetic parameters (kon, koff) and resulting (overall) KD of monovalent interactions determined for B2G variants and corresponding parental antibodies were subsequently presented in ‘on/off rate plots’ for comprehensive comparisons.

### 2.4. CD138 Avidity SPR

For determining avidity-driven binding kinetics to CD138, the antigen was immobilized to 240 response units (RU) on a CM5 chip using the amine coupling kit (GE Healthcare) at pH 4.5 and 1 µg/mL. Thereafter, the interaction of CD138 on the CM5 surface and anti-CD138 antibody affinity variants was analyzed in a dilution series of 33 nM to 300 nM in HBS-P+ (GE Healthcare) using 120-s association time and 600-s dissociation time at a flow rate of 60 µL/min. The surface regeneration was performed by a 60-s washing step with a 3 M MgCl2. All Biacore T200 experiments were carried out in HBS-P+ (GE Healthcare) pH 7.4 running buffer and at 25 °C. Binding curves were evaluated using Biacore T200 evaluation software. For the calculation of binding properties, a 1:1 Langmuir binding model was used. Further analysis of a heterogenous interaction was performed using Interaction Map software (Ridgeview Instruments AB). All kinetic parameters (kon, koff) and resulting (overall) KD of monovalent interactions determined for B2G variants and corresponding parental antibodies were subsequently presented in ‘on/off rate plots’ for comprehensive comparisons.

### 2.5. Her2 Affinity SPR

The anti-Her2 antibody affinity variants at 5 nM in HBS-P+ (GE Healthcare) were captured with anti-hFc antibody (GE Healthcare BR-1008-39) on a CM5 sensor chip for 30 s (capture Level ~ 100 RU). Thereafter, huHer2 ECD (In house) was injected at a concentration of 19 nM to 300 nM diluted in HBS-P+ (GE Healthcare) with 150-s association time and 720-s dissociation time. All Biacore T200 experiments were carried out in HBS-P+ (GE Healthcare) pH 7.4 running buffer at 25 °C. Binding curves were evaluated using T200 evaluation software. For the calculation of binding properties, a 1:1 Langmuir binding model was used. All kinetic parameters (kon, koff) and resulting (overall) KD of monovalent interactions determined for B2G variants and corresponding parental antibodies were subsequently presented in ‘on/off rate plots’ for comprehensive comparisons.

### 2.6. Her2 Avidity SPR

For determining avidity-driven binding kinetics, the huHer2 ECD (In house) was immobilized to 60 response units (RU) on a CM5 chip using the amine coupling kit (GE Healthcare) at pH 4.5 and 1 µL/min. Thereafter, the interaction of huHer2 ECD on the surface and anti-hHer2 antibody variants was analyzed in a dilution series from 7 nM to 600 nM in HBS-P+ (GE Healthcare) using 150-s association time and 600-s dissociation time at a flow rate of 60 µL/min followed by regeneration of the surface by a 60-s washing step with 3 M MgCl2. All Biacore T200 experiments were carried out in HBS-P+ (GE Healthcare) pH 7.4 running buffer and at 25 °C. Binding curves were evaluated using Biacore T200 evaluation software. For the calculation of binding properties, a 1:1 Langmuir binding model was used. All kinetic parameters (kon, koff) and resulting (overall) KD of monovalent interactions determined for B2G variants and corresponding parental antibodies were subsequently presented in ‘on/off rate plots’ for comprehensive comparisons.

### 2.7. EGFR Affinity SPR

The anti-EGFR antibody affinity variants at 5 nM in HBS-P+ (GE Healthcare) were captured with anti-hFc antibody (GE Healthcare BR-1008-39) on a CM5 sensor chip for 30 s (capture Level ~ 100 RU). Thereafter, the interaction with the huEGFR ECD antigen (In house) was analyzed in a dilution series from 19 nM to 300 nM using 120-s association time and 600-s dissociation time at a flow rate of 60 µL/min. All Biacore T200 experiments were carried out in HBS-P+ (GE Healthcare) pH 7.4 running buffer at 25 °C. Binding curves were evaluated using Biacore T200 evaluation software. For calculating binding properties, a 1:1 Langmuir binding model was used. All kinetic parameters (kon, koff) and resulting (overall) KD of monovalent interactions determined for B2G variants and corresponding parental antibodies were subsequently presented in ‘on/off rate plots’ for comprehensive comparisons.

### 2.8. EGFR Avidity SPR

An SA-chip was coated with ~200 response units (RU) of hEGFR ECD antigen (In house). Binding kinetics were determined using a dilution series from 1.1 nM to 90 nM anti-hEGFR antibodies in HBS-P+ (GE Healthcare) and a flow rate of 60 µL/min with a 120-s association time and 600-s dissociation time. The surface regeneration was performed by a 30-s injection of 10 mM NaOH. All Biacore T200 experiments were carried out in HBS-P+ (GE Healthcare) pH 7.4 running buffer at 25 °C. Binding curves were evaluated using Biacore T200 evaluation software. For calculating the binding properties, a 1:1 Langmuir binding model was used. Further analysis of the heterogenous interaction was performed using Interaction Map software (Ridgeview Instruments AB). All kinetic parameters (kon, koff) and resulting (overall) KD of monovalent interactions determined for B2G variants and corresponding parental antibodies were subsequently presented in ‘on/off rate plots’ for comprehensive comparisons.

### 2.9. Poly-Reactivity Assays

ELISA-based poly-reactivity assays were performed using non-specific antigens and specific antigens (human Syndecan-1, R&D-Systems, 2780-TS, huHer2 ECD, in house CC-13-13, and huEGFR ECD, in house P1AD7166) serving as positive controls. The antigens were coated to 384-well Nunc-MaxiSorp plates at concentrations between 0.1 and 2 µg/mL in PBS at 4 °C overnight. Between individual steps, plates were washed with PBST (1×PBS + 0.1% Tween 20). Blocking was performed (2% BSA in PBS + 0.2% Tween 20) for 1 h at room temperature without agitation. Antibody samples (1 µg/mL in One Step ELISA buffer, 1×PBS + 0.5% BSA + 0.05% Tween 20) were incubated 1 h at room temperature without agitation. Secondary antibody (anti-human IgG Fc-specific, Jackson #109-036-098, dilution 1:7000 in OSEP) was added (1 h at room temperature on a microplate shaker at 400 rpm). A detection substrate TMB was added (4 min without agitation) and absorbance was measured at a wavelength of 370 nm and a reference wavelength at 492 nm using a Tecan Safire II ELISA reader. The raw measurement signals at 492 nm were subtracted from the respective raw measurement signals at 370 nm (absorbance correction). The absorbance corrected signals from the blank wells (secondary antibody only) were subtracted from the absorbance corrected signals on the respective antigens (Blank correction). All measurements were done in triplicates.

### 2.10. Computational Analysis

The set of X-ray crystal structures used to generate the antibody-antigen contact statistics consisted of 521 antibody-protein complexes and 205 antibody peptide complexes, which are non-redundant with regard to the CDR sequences of the antibodies involved. All structures were retrieved from the structural antibody database SAbDab [30]. The dataset was divided by antigen type (protein or peptide) since the antigen type has an impact on the preferential paratope residues and, thus, leads to different contact statistics. Structural parameters investigated to quantify antibody-antigen contact propensity for each residue in the Fv were the average sidechain solvent accessibility change (antigen present versus antigen absent) and the average number of chemical interactions with the antigen. All structural analyses were performed with BIOVIA Discovery Studio [31] and Pipeline Pilot [32]. ANARCI assigned all numbers of the antibody sequences used in this study [33].

## 3. Results

### 3.1. The B2G (Back-To-Germline) Procedure

The procedure defines affinity-modulating back-mutations for any given animal-derived or human- derived VH or VL protein sequence. It ‘reverses,’ in part, the antibody maturation process in B-cells (see Figure 1A) and can, hence, not be applied to antibodies generated from in-vitro library panning/selection approaches of (synthetic) libraries. The procedure bases on CDR sequences of original antibodies (animal-derived prior to potential humanization) and converts those back to the germline of the organism from which the original antibody was derived. Residues that are converted to the ‘animal germline’ are those identified to have a high likelihood to interact with antigens. In this regard, this procedure is ‘the opposite’ of super-humanization (super-humanization would convert those non-human CDR residues to the human germline, which have a low propensity to contribute to antigen contacts). Applying protein sequences of the variable regions of a given antibody as input, the procedure consists of three subsequent steps. An overview of the procedure is shown in Figure 1B, details are provided in the material and method section.

### 3.2. The First Step Identifies Germlines from Which the Input Antibody Might Be Derived

The VH and VL germlines from which the antibody might have descended are identified by performing a BLASTp [34] search against a custom database of the IGHV and IGKV/IGLV germline sequences obtained from the IMGT [35,36]. For both VH and VL, the hit with the highest BLASTp percent identity is assumed to be the germline of origin. Due to the complexities of VDJ recombination in combination with the occurrence of additional mutations in the process of maturation, it is not possible to determine the definitive non-matured (VDJ only) protein sequence of CDR-H3 [37]. It is, therefore, not considered any further at this stage.

The search can be narrowed down by specifying the species of origin of the antibody. In cases where the input sequences belong to a humanized antibody and the species of origin is unknown, it is not trivial to identify the non-human germline that brought forth the original CDRs. To increase the chance of finding the correct IGHV germline of the species of origin, the database is queried with the sequence segment ranging from the beginning of CDR-H1 to the end of CDR-H2 only. This reduces the bias by irrelevant framework regions and eliminates the obfuscation caused by the hypervariable sequence of CDR-H3. This procedure is not necessary for IGKV or IGLV: As CDR-L3 is encoded by the V gene, the sequence typically contains enough information to retrieve the correct germline of origin, even when the framework regions have been humanized.

After the most likely germline sequences have been identified, they are aligned to the input sequences based on their WolfGuy [38] numbering. Every mismatch between input and germline sequence represents a possible maturation event, and, thus, a potential candidate for a B2G back-mutation.

### 3.3. The 2nd Step Is the Selection of the Most Promising B2G Back-Mutation Candidates among All Matured Positions

In order to select B2G back-mutations with a high probability of being involved in antigen binding, the following criteria are applied: (i) The amino acid to back-mutate must be situated in the CDR regions or in the framework positions directly preceding CDR-H3 (WolfGuy 331 and 332). The final positions of CDR-H2 (WolfGuy 295 to 299) are always excluded. (ii) The amino acid side chain should not be completely buried. (iii) The amino acid side chain should not be involved in VH-VL interactions. (iv) There are known antibody-antigen complex structures in the PDB [39] where the amino acid side chain at this position is involved in chemical interactions with the antigen.

The structural read-out necessary for criteria (ii) and (iii) is extracted from a homology model of the variable region of the antibody, which, in our case, is generated with MoFvAb [40]. Homology models of the antibody variable region generally achieve a very high level of accuracy in all regions except CDR-H3 [41]. For the B2G approach, the accuracy of the CDR-H3 loop modeling is only of minor relevance since CDR-H3 is exempt from B2G mutations. The statistics about antibody-antigen contacts used for (iv) are extracted from a set of antibody complex structures. The parameters chosen for defining the likelihood of antigen contact include solvent accessibility changes and chemical antigen interactions, as described in detail in the Data S1 section, including a table listing the predicted antigen contact parameters for each CDR residue in VH and VL. Alternatively, one could also predict the likelihood of individual residues to be involved in antigen binding with a paratope predictor such as ParaPred [42]. 

### 3.4. The Third Step Is the Stratification of Selected B2G Back-Mutations Based on Their Predicted Impact on Antigen Binding

To obtain, per input sequence, multiple B2G variants with an increasing degree of devolution, B2G mutations are stacked up in a step-wise process that is applying criterion (iv), i.e., the average number of antigen interactions in known complex structures, with certain thresholds. For example, in the initial B2G variant of VL, all positions with an average of more than 0.01 and less than 0.25 antigen interactions are back-mutated to germline, which aim at a very moderate loss of binding affinity. In the next variant, this threshold is raised to 0.5 antigen interactions, which leads to a growing number of back-mutated positions and an increasing probability of loss of binding affinity. The emerging B2G sequence variants are checked for N-glycosylation sites that might have been introduced in the process. Any B2G mutation that has created a new N-glycosylation site is discarded and the original amino acid is restored. Duplicate sequences are removed. It is possible to additionally implement alanine replacement at an interaction-prone position of CDR-H3 (i.e., at positions that are not amenable to B2G) to further increase the potential for significant loss of binding affinity (those changes would generate V-regions that are B2G: Ala-replacement hybrids).

The final output of the procedure is as a set of variants of VH and VL sequences of the input antibody. Combinations of those sequences define antibody variants with different degrees of devolution, i.e., molecules that may have just one matured position replaced by the original germline residue, molecules with several germline replacements in VH, and/or VL up to molecules that carry all CDRs of VL as well as CDR-H1 and CDR-H2 in the germline configuration.

### 3.5. B2G Variants of Different Antibodies

To demonstrate that the above described procedure is suited to generate affinity-modulated variants, the B2G procedure was applied to three different antibodies with different specificities: <CD138> (US9446146B2), <Her2/neu> (Trastuzumab, US6870034B2, [26], <EGFR> (Cetuximab, US6217866B1). The ‘wild type’ sequences of these antibodies were fed into the B2G procedure as described above to define VH and VL back-mutations for each V sequence. Those were subsequently combined as VL-VH pairs to generate antibody variants. These B2G variants (we selected those with high antigen contact score for each chain, defined by Kabat positions [43]) and the resulting VH-VL combinations that we chose to generate affinity-modulated V regions, as well as the denominations, identifiers, and combinations for individual V-regions, are listed in Table 1.

### 3.6. Generation of B2G-Variants 

The antibody variants that are listed in Table 1 were produced as humanized IgG1 molecules via CMV-promoter driven transient expression in HEK293F or HEK-Expi cells, as previously described [27,28] (see the Section 2 for details). The recombinant antibodies that were secreted into cell culture supernatants were subsequently purified by identical means as their parent antibodies, by applying Protein A affinity chromatography followed by size-exclusion chromatography (SEC) (see Section 2). All B2G variants showed a ‘benign’ behavior with low propensity to aggregate and complete compatibility with standard purification procedures. All antibody derivatives could be purified to homogeneity, as demonstrated by analytical SEC and SDS-PAGE (see Figure S1 for details). Expression yields of the antibody variants were similar to those of their respective parent antibodies, in the range of 50–300 mg/L culture supernatant dependent on the parental antibody from which the variants were derived, and dependent on the expression system (HEK-Expi generated higher yields than HEK293F in our hands). The final yields of all antibodies and derivatives following Protein A and SEC are listed in Table S1. Correct composition and identity of each produced antibody additionally verified by electrospray ionization mass spectroscopy (Section 2 and Figure S1). None out of the 28 produced B2G variants was associated with unusual/deviant properties or problems during expression, purification, and further handling. This indicates that the introduction of B2G mutations into Fv does not significantly alter biophysical or expression properties of parent antibodies. 

### 3.7. B2G Variants Retain Specificity to Cognate Antigen in a Wide Affinity Range

**Figure 2:**
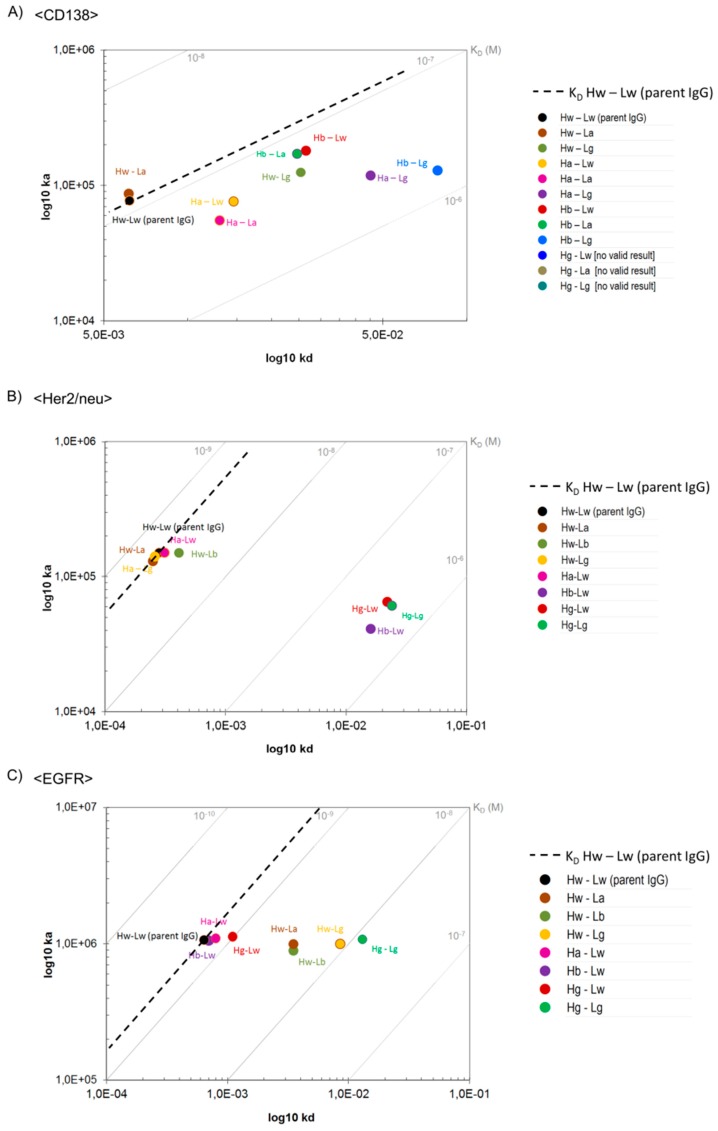
On-/Off-rate plots showing monovalent binding of B2GL variants to antigens. Surface Plasmon Resonance was applied to measure differences in binding kinetics of (**A**) <CD138>, (**B**) <Her2/neu>, and (**C**) <EGFR> variants. As a reference, note the dashed line indicating K_D_ values of parental IgGs.

Surface Plasmon Resonance (SPR) with monomeric antigens as analytes (to assess monovalent binding) was applied to determine the effect of B2G mutations on affinity to cognate antigens (details in the Section 2). The on-rates and off-rates and dissociation constants (K_D_) of the variable regions of B2G derivatives that bind CD138, Her2/neu, or EGFR in comparison to their parent antibodies are shown in Figure 2. These analyses revealed that the B2G procedure generated variants of all specificities (CD138, Her2/neu, and EGFR), which results in diverse sets of antibodies that retained specificity yet with different affinities. The mutations that we introduced to generate these antibodies affected on-rates as well as off-rates (Figure 2). On-rate modulation was observed for some CD138 binders, some displaying increased and others displaying decreased on-rates (Figure 2A). Similarly, B2G derivatives of <Her2/neu> either retained or slowed the on-reactions (Figure 2B). In contrast, B2G variants of EGFR-binders did not display significant alterations in their on-rates (Figure 2C). Off-rate modulating B2G variants (i.e., antibodies with enhanced dissociation and, in consequence, lower affinity) were obtained for all three antibodies. 

**Figure 3:**
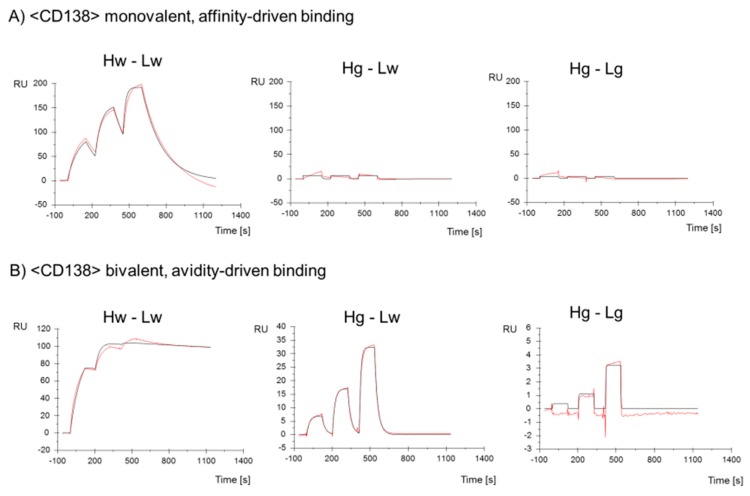
Binding of parent <CD138> and -B2G variants to the hCD138 antigen. Surface Plasmon Resonance with monomeric CD138 protein as analyte assesses (**A**) monovalent and (**B**) bivalent binding. SPR profiles of Hg-Lw and Hg-Lg variants show a weak remaining resonance when analyzed in the bivalent assay.

The K_D_ (k_a_/k_d_) of all antibody derivatives always showed either the same/similar, or a reduced affinity compared to parent molecules (Figure 2 and Table S2). Even B2G variants that displayed increased on-rates bound their respective antigens with reduced affinity (K_D_) as in those faster on-rates, which were overcompensated by even faster off-rates (e.g., CD138 Hb-Lg antibody, Figure 2A). Predominantly reduced affinity and lack of increased affinity is not unexpected considering that the B2G procedure reverses affinity-increasing maturation events. The influence of different B2G mutations and combinations of on-rates and off-rates (and on resulting K_D_) is exemplarily shown as SPR-profiles for B2G variants of <CD138> in Figure 3. The SPR data for all other binders are provided in Figures S2 and S3.

The B2G family of <CD138> whose parent binds with a K_D_ of 1.0 × 10^−7^ M includes a variant with similar affinity (e.g., Hw-La) (Figure 2A) and variants with intermediate loss of affinity (e.g., Hb-La, Hw-Lg) (Figure 2A). Germline mutations in the light chain reduced the affinity by approximately nine-fold (e.g., Hb-Lg,) and variants that have CDR1 and CDR2 of the H chain reverted to germline such as Hg-Lw and Hg-La showed a more than 100-fold reduced affinity. B2G derivatives of <Her2/neu> whose parent binds with a K_D_ of 1.9 × 10^−9^ M includes variants with the same/similar affinity (e.g., Hw-La, Ha-Lg) and antibodies with more than a 200-fold reduced affinity (H chain mutations), such as Hb-Lw, Hg-Lw, and Hg-Lg (Figure 2 and Table S2). Lastly, the family of B2G members of <EGFR> whose parent binds with a K_D_ of 6.0 × 10^−10^ M includes variants with the same/similar affinity (e.g., Hb-Lw, Ha-Lw), variants with an intermediate loss of affinity (e.g., Hw-La, Hw-Lb), and antibodies with a 20-fold reduced affinity (e.g., variants that carry mutations in CDR1, 2, and 3 of the light chain) such as Hw-Lg and Hg-Lg (Figure 2 and Table S2). 

### 3.8. B2G Generates Antibody Variants That Depend on Avidity for Effective Binding

**Figure 4:**
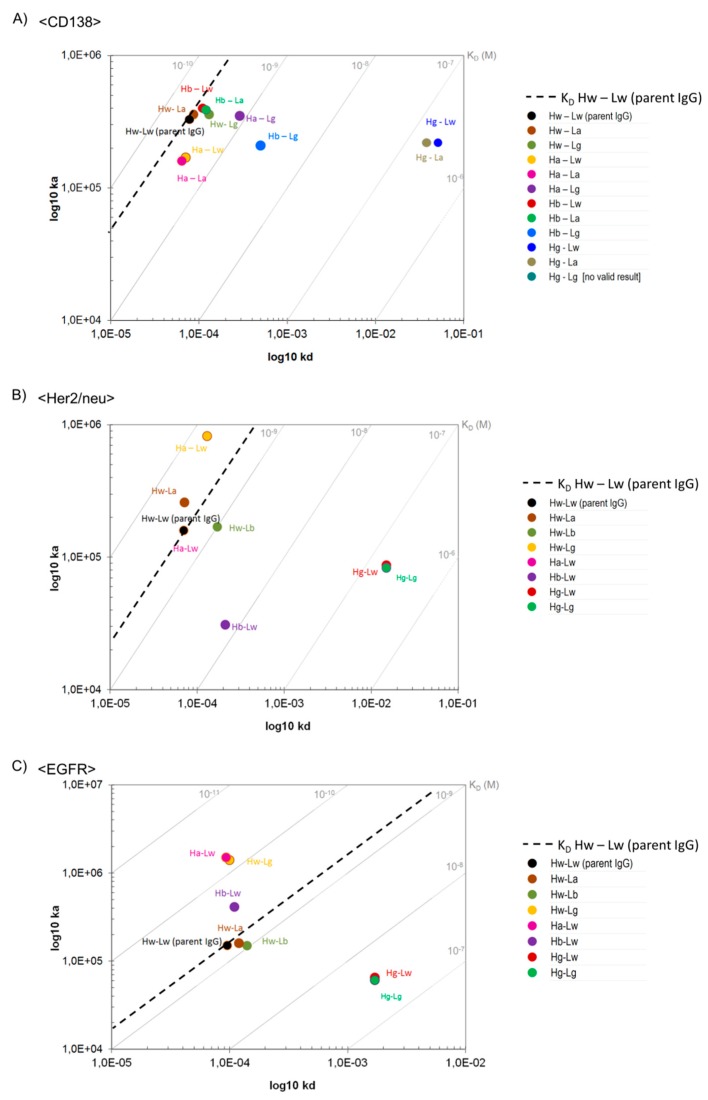
On-/Off-rate plots showing bivalent binding of B2GL variants to antigens. Surface Plasmon Resonance was applied to measure differences in binding kinetics of (**A**) <CD138>, (**B**) <Her2/neu>, and (**C**) <EGFR> variants. As a reference, note the dashed line indicating K_D_ values of parental IgGs.

The ‘ideal concept’ of avidity-enhanced specificity requires antibodies that do not bind/retain the antigen in a monovalent manner yet show antigen binding in avidity settings. In consequence, SPR analyses with monomeric antigens as analytes would indicate a complete lack of specific binding. The same antibodies, however, should display specific antigen binding in SPR assays that cover avidity via bivalent antigen contacts. Figure 3 shows that the B2G procedure is suitable to generate antibodies with those characteristics. An example for an antibody that requires avidity for effective binding is the CD138-binding antibody Hg-Lw for which antigen binding cannot be detected/evaluated by SPR when immobilizing the IgG and applying monomeric antigens as analytes (Figure 3A). The only hint toward potentially very weak interactions under those assay conditions are slight increases of ‘background’ signals upon exposure to analyte, which are absent in completely negative controls derived from the same antibody (compare Hg-Lw and Hg-Lg in Figure 3A). Addressing the same antibody in an avidity binding assay (Figure 3B), i.e., by inverting the SPR setup (high density immobilization of the target antigen followed by probing with bivalent IgG), specific binding can be detected with ‘normal’ on-rates and fast off-rates (Figure 3B and Figure 4A, Hg-Lw). Other examples of antibody variants that fulfill or come close to binding properties desired for avidity-enhanced binding are the light chain germline back mutated Hw-Lb or Hw-Lg variants of EGFR, which bind monovalent with very low affinity (K_D_ 3.9 × 10^−9^ or 8.5 × 10^−9^ M) but retain high affinity binding properties via avidity (K_D_ 8.0 × 10^−10^ and 1.0 × 10^−10^ M respectively) (Figure 4C and Table S3). The results for avidity-driven affinity measurements of parent and B2G derivatives of all antibodies are shown in Figure 4 while the details are listed in Table S3).

### 3.9. B2G Variants Retain Specificity without Introducing Polyreactivity

One concern that needs to be addressed when mutating antibody Fv regions toward reduced affinity is that this may affect specificity. In particular, general stickiness or polyreactivity may be introduced by such alterations in which examples previously reported for antibodies with reduced affinities were introduced by alanine replacement [15,16]. Therefore, poly-reactivity assessments were performed by exposing parent and mutated antibodies to a diverse panel of unrelated antigens including defined proteins such as cardiolipin, heparin, parathyroid hormone, DNA, hemocyanin, streptavidin, BSA, HSP70, insulin, gelatin, albumin, histone, and whole cell lysates derived from *E. coli*, as previously described [44,45,46,47,48,49] (see assay details in the M&M section). The cognate antigens were simultaneously detected in the same ELISA assays to serve as positive controls.

**Figure 5:**
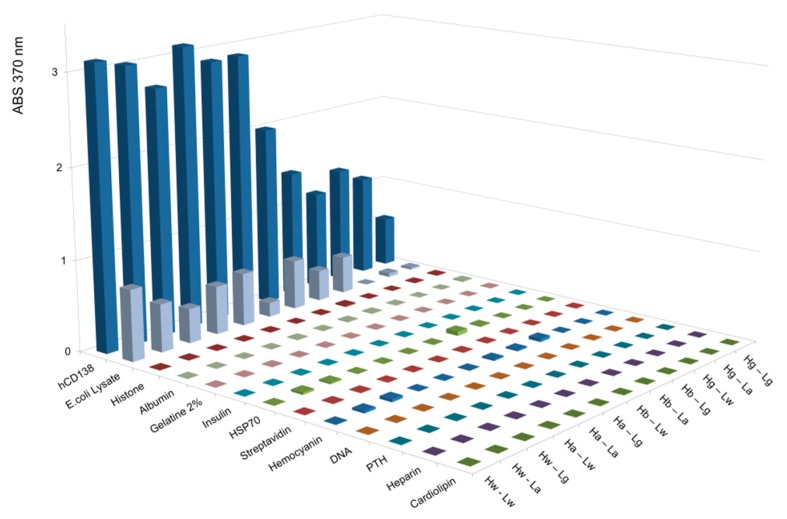
ELISA-based poly-reactivity assessment of parental <CD138> IgG and B2GL variants. Poly-reactivity for indicated variants was assessed using non-specific antigens and specific antigen (human Syndecan-1, R&D-Systems, 2780-TS) as a positive control. The B2G variants of <CD138> do not elicit increased or additional nonspecific signals when compared to the parental IgG Hw-Lw. PTH = Parathyroid hormone.

The results of these analyses reveal that affinity reduction via B2G does not affect the specificity and does not introduce poly-reactivity. The parent CD138 binder, for example (Figure 5), shows strong reactivity to its cognate antigen but no detectable reactivity to the other probes, with the exception of weak signals upon exposure to *E. coli* lysates. In a similar manner, B2G variants of <CD138> do not elicit increased or additional nonspecific signals (in fact, some show reduced binding to E. coli extract compared to the parent antibody). B2G variants of <Her2/neu> and <EGFR> (Figure S4) and did also not generate increased or additional nonspecific signals in poly-reactivity assessments. Similarly, lack of poly-reactivity was also observed for antibody variants that harbored alanine at positions defined by B2G (see below and Figure S5).

Thus, B2G mediated the reversion of maturation processes generating antibodies with reduced affinity, which retain their specificity without the introduction of poly-reactivity.

### 3.10. Comparison of B2G with Alanine Replacement 

**Figure 6:**
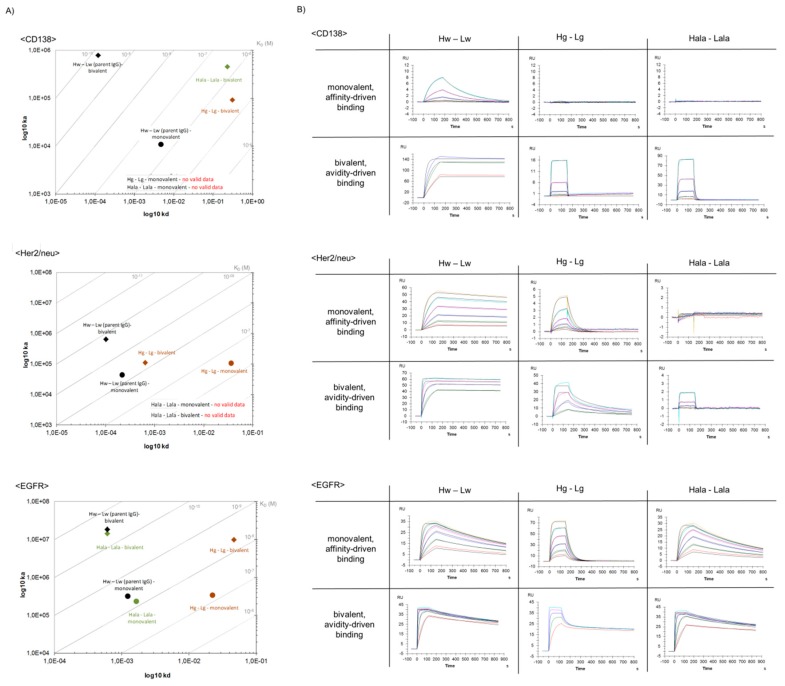
Comparison of binding kinetics and SPR profiles of B2GL variants vs. alanine replacement variants. Shown are (**A**) on-/off-rate plots and (**B**) SPR profiles based on affinity-mediated and avidity-mediated binding kinetics.

The currently, most frequently, applied method to modulate affinity of antibodies is the replacement of CDR residues with alanine (AlaR). Positions for replacement are defined either by random scanning or by structure-based choices [50,51,52]. To compare the B2G and AlaR procedures, a set of antibodies was generated, which harbored alanine instead of germline residues at the positions that deviated from parent antibodies (Table 1 and Table S4). A comparison of the binding characteristics of those antibodies with corresponding parent and B2G-derived antibodies is shown in Figure 6.

Interestingly (and dependent on the individual modified antibody), B2G and AlaR resulted in two of three examples in antibodies with different properties. The B2G-derived and AlaR-derived CD138 binders showed similar binding properties. Both showed strongly reduced binding compared to the parent antibody (with negligible monovalent and unambiguous bivalent binding). In contrast to that, divergent properties were observed for Her2/neu binders. Affinities of B2G–derivatives were reduced compared to parent IgG but still capable to bind in a monovalent as well as bivalent assay setting. Alanine replacement at the same positions, however, abrogated binding to Her2/neu (completely in monovalent and reduced to very weak/not detectable in avidity assays).

EGFR-binding antibodies showed divergent properties when comparing B2G-derived and AlaR-derived variants in an inverse direction, as observed for Her2/neu-binders. B2G–derivatives showed significantly reduced affinities compared to parent IgG while AlaR generated variants that retained most of the affinity of the parent antibody. Poly-reactivity assays performed in the same manner, as described in Figure 5, revealed low poly-reactivity for AlaR variants in the same manner as described above for B2G variants (Figure S5).

In summary, our data indicate that both approaches can be applied to modulate the affinity. B2G, however, may be more reliable if one aims to generate a set of antibodies that retain specificity (without poly-reactivity) and covers a wide range of reduced affinities.

## 4. Discussion 

*The B2G procedure* reverts antibody maturation events by replacing residues that were generated by somatic mutation with corresponding original germline residues. B2G alters only residues that can unambiguously be defined as mutation-derived. In consequence, B2G can be applied to all animal/human-derived L-chain CDRs and to CDR1 and CDR2 of H-chains.

In case of antibodies that carry many somatic mutations in their CDRs, the number of B2G candidates can be reduced by defining preferred choices for germline replacements and -combinations. Additionally, in nature, somatic hypermutation (SHM) in B cells does not necessarily lead to increased affinity [53]. The highest affinity cells are selected through the ability to acquire antigen from the surface of follicular dendritic cells and present this antigen to T follicular helper cells. Therefore, the likelihood of antigen contact, in this case, is determined for each residue deviating from the germline, and the possible options are ranked and selected accordingly.

The automatic selection procedure that we implemented uses certain structural properties (e.g., solvent accessibility) that are extracted from a homology model of the input antibody. The likelihood of a given residue to form chemical interactions with the antigen of a given type is estimated from a database of antibody complex structures [39]. While the procedure relies on structural data, it does not require an expert review of the input structure. Alternatively, one could also envision a completely sequence-based B2G selection procedure where the average solvent-accessible surface area and other structural features of a given residue are estimated from a pre-processed structural database, analogously to the statistics regarding the likelihood of antibody-antigen interactions.

The generation of CDR-H3 is initiated by a VDJ-rearrangement. Subsequently, somatic hypermutation are set upon this already re-arranged CDR [37]. The initial VDJ step that forms the template for maturation at this CDR is too complex to unambiguously call underlying germline sequences. This also prevents the differentiation of original and matured sequences in this region. Therefore, we cannot apply B2G to change CDR-H3 regions. In consequence, B2G-derived antibodies with the highest degree of de-maturation harbor germline sequences in all L-chain CDRs as well as CDR1 and 2 of the H-chains, but retain H-chain CDR3 of the parent antibody.

Even though it is impossible to differentiate mere VDJ-sequences from those with additional maturation events, it is still possible to modulate binding contributions of CDR-H3 by mutating key residues. One option to implement this would be to select interaction-prone residue in CDR-H3 and replace them with alanine. Alternatively, residues at those positions may be replaced with residues that are most frequently found at this position in antibody populations. Such extended B2G hybrid approaches may generate additional antibodies with modulated affinities. We observed, however, that ‘clean’ B2G procedures that retain CDR-H3 are already sufficient to convert parent antibodies to derivatives that cover large ranges of affinities.

Assuming that early antibodies must be antigen-specific to undergo maturation, all variants generated by B2G should be specific. The fact that B2G variants recapitulate such naturally occurring antibodies may explain that all variants that we analyzed (even the low affinity variants) did not increase nonspecific stickiness or poly-reactivity when compared to parent antibodies. The generation of ‘natural’ variants is a conceptual difference compared to other affinity modulation approaches. Alanine replacements or CDR-mutations, which do not re-generate de-matured yet specific variants, carry a risk of introducing poly-reactivity [15,16]. A comparison of B2G and AlaR approaches revealed that B2G-mediated reduction of affinity does not only prevent poly-reactivity, but also leads to more predictable affinity modulation. We observed mutations of the same positions with alanine instead of germline residues, which modulate affinity in a rather unpredictable manner. Affinities of antibodies generated by AlaR ranged from no effect on affinity (ineffective) to gradually decreased affinity (desired) to complete abrogation (not desired) of binding. In contrast, B2G reliably generated for the same parent antibody sets of variants with increasingly reduced affinities upon increased degree of de-maturation.

*Effects on antibody affinity of B2G-defined mutations that trigger CDR maturation* can be assessed by comparing on-rates and off-rates of derivatives that represent different stages of maturation. With few exceptions, de-maturation altered the affinity compared to parent antibodies, which influenced on-rates as well as off-rates. All de-matured antibodies whose affinity diverged from parent counterparts displayed reduced affinity. This is expected as the antibody maturation aims at generating/selecting ‘better’ antibodies represented by increased affinities. An accelerated off-rate of B2G variants was the predominant cause for reduced affinity and improved off-rates were not observed for any variant. Interestingly (and in contrast to off-rates), B2G variants were altered not only toward slower on-rates, but, in some instances, also toward faster on-rates. In the latter case, antibodies that were closer to germline presented with faster on-rates (usually triggering increased affinity). Those improved on-rates were, however, in all cases accompanied with even faster off-rates, with overall affinities (KD’s) of de-matured antibodies worse than those of parent antibodies. An explanation for decreased on-rates (overcompensated by increased off-rates) may be that maturation generates more tight or defined binding pockets. Initial non-matured binding sites facilitate antigen contact with binding sites upon their first encounter with the antibody. Antigens may encounter other antigens in imperfect orientations yet still be able to accommodate and attach those triggered by first contacts of anchor residues. The disadvantage of such interactions is that they are also prone to lose contact quite rapidly. More matured binding sites will provide better fits or contacts with the antigen (improved off-rate), yet with higher probability of antigens to ‘bounce off’ (decreased on-rate) when encountering antibodies in unfavorable orientations [25]. The observation that a higher degree of maturation correlated with increased affinity, even if accompanied with slower on-rates, may indicate that maturation selects antibodies with improved off-rates over those that retain or increase on-rates.

*Bispecific antibodies with avidity-enhanced specificity* require combinations of binding modules that enable specific binding to surfaces that are bound by both entities without binding to surfaces that can only be bound by one entity. Those binding modules can be generated by the B2G procedure since it converts parent antibodies to sets of derivatives with different affinities. The fact that B2G generates variants that have no only off-rates but also on-rates modulated is particularly useful for generation of such bsAbs. Avidity-enhanced specificity can be achieved by combining reduced affinity binders of fast-on/fast-off and slow-on/slow-off types. Non-target tissues that, with only one accessible antigen, are either only transiently bound but not retained by the fast-on/fast-off arm, or do not attach in the first place if the expressed antigen is the target of the slow-on/slow-off entity. On cells or tissues that display both antigens, however, bsAbs are captured by the fast-on/fast-off entity and become ‘fixed’ by the delayed binding slow-on/slow-off entity. For the examples that we analyzed, B2G delivered both types of desired antibody variants (fast-on/fast-off as well as slow-on/slow-off) while alanine replacement modulated predominantly off-rates.

The B2G procedure generates antibody derivatives with different (reduced) affinities to given antigens to be used as building blocks for bsAbs that simultaneously address different antigens on the surfaces of cells. This procedure can be applied to any (animal-derived) antibody but can only address the first requirement for such dual-targeting bsAbs. Simultaneous attachment to cell surfaces requires compatible antigen densities, geometries, and flexibilities and possibly internalization parameters. Those parameters will be different for each antigen/-combinations and possibly for paratope combinations. Because of that, those parameters cannot be addressed by a general platform such as B2G. B2G provides the first step in generating such bsAbs but must be accompanied by compatibility assessments for combinations of antigens/paratopes and binders. 

Among the B2G family of <CD138> binders, we identified variants (e.g., germline mutations in the light chain) showing a change of affinity in the same magnitude (~10 fold) as observed when comparing affinities of primary and secondary response antibodies isolated from C57BL/6 mice [54]. Furthermore, the range of affinities generated via B2GL variants was comparable to the range of equilibrium constants (10^−7^–10^−10^ M) measured with antibodies generated through hybridoma technology [55]. These results suggest that the introduced mutations in CDRs of H and L chains via B2GL lead to an alteration of affinity that correlates inversely to natural maturation events in B cells and that the B2GL-approach is a useful tool to predict and generate low-affinity binders from existing high affinity parental IgGs.

## Figures and Tables

**Table 1:** B2G variants in VH and VL of different antibodies. Positions are defined in accordance with the Kabat numbering scheme. * ‘germline’ of VH can be defined only for CDR1 and CDR2 because H-chain VDJ rearrangement is too complex to unambiguously call the composition of the original VDJ-event and to differentiate that from potential additional hypermutations placed on top of that. Sequences that do not deviate from the original input antibody are termed Hw and Lw (‘wild-type; parental IgG’), Sequences that have CDRs completely reverted to germline (except for H-CDR3) are termed Lg and Hg* (‘germline’). Sequences that have those CDR positions (of Lg and Hg) mutated to alanine are termed Hala-Lala.

	Identifier	CDR1	CDR2	CDR3	VH + VL Combinations
<CD138> VH	Hw	-	-	-	Hw-Lw (parent IgG) Hw-La Hw-Lg Ha-Lw Ha-La Ha-Lg Hb-Lw Hb-La Hb-Lg Hg-Lw Hg-La Hg-Lg Hala-Lala
	Ha	-	H56 R→S	-	
	Hb	H30 S→T H31 N→G	-	-	
	Hg *	H30 S→T H31 N→G	H54 T→S H56 R→S H58 I→N	-	
<CD138> VL	Lw	-	-	-	
	La	-	L55 Q→H	-	
	Lg	L30 N→S	L53 T→S L55 Q→H	-	
<EGFR> VH	Hw	-	-	-	Hw-Lw (parent IgG) Hw-La Hw-Lb Hw-Lg Ha-Lw Hb-Lw Hg-Lw Hg-Lg Hala-Lala
	Ha	H31 N→S	-	-	
	Hb		H56 N→S	-	
	Hg *	H31 N→S	H56 N→S	-	
<EGFR> VL	Lw	-	-	-	
	La	L32 N→S	-	-	
	Lb	-	-	L91→S L93→S	
	Lg	L32 N→S	-	L91→S L93→S	
<Her2/neu> VH	Hw	-	-	-	Hw-Lw (parent IgG) Hw-La Hw-Lb Hw-Lg Ha-Lw Hb-Lw Hg-Lw Hg-Lg Hala-Lala
	Ha	H34 I→M	-	-	
	Hb	-	H52 Y→D H53 T→A H56 Y→N H58 R→K	-	
	Hg *	H34 I→M	H52 Y→D H53 T→A H56 Y→N H58 R→K	-	
<Her2/neu> VL	Lw	-	-	-	
	La	L30 N→S	-	-	
	Lb	L30 N→S	L53 F→Y	L93 T→S	
	Lg	L24 R→K L30 N→S	L53 F→Y L54 L→R L56 S→T	L93 T→S	

## Supplementary Materials

The following are available online at https://www.mdpi.com/2073-4468/8/3/45/s1, Figure S1: Analytical verification of B2G variants, Figure S2: SPR Plots of all affinity measurements, Figure S3: SPR Plots of all Avidity measurements, Figure S4: Polyreactivity assays of <Her2/neu> and <EGFR> B2G variants, Figure S5: Polyreactivity assays of <CD138> variants, Table S1: Expression rates of antibodies and their B2G variants, Table S2: Affinity modulation by B2G, Table S3: Avidity modulation by B2G, Table S4: Expression yields of antibody derivatives that harbor alanine at B2G-defined CDR positions, Data S1: Differentiation of CDR residues by addressing probability of antigen interaction.
