# Supplementary material for: Back-to-Germline (B2G) Procedure for Antibody Devolution
Source: Antibodies (Basel). 2019 Aug 26;8(3):45. doi: 10.3390/antib8030045 (PMC6784197; doi:10.3390/antib8030045)
Supplement: Supplementary file 1 [file antibodies-08-00045-s001.pdf]

antibodies

Supplemental Data

Back-to-germline (B2G) procedure for antibody devolution

Anja Schrade, Alexander Bujotzek, Christian Spick, Martina Wagner, Johannes Goerl, Xenia Wezler, Guy Georges, Roland Kontermann and Ulrich Brinkmann

Suppl. Data S1: Expression and Purification of B2G variants

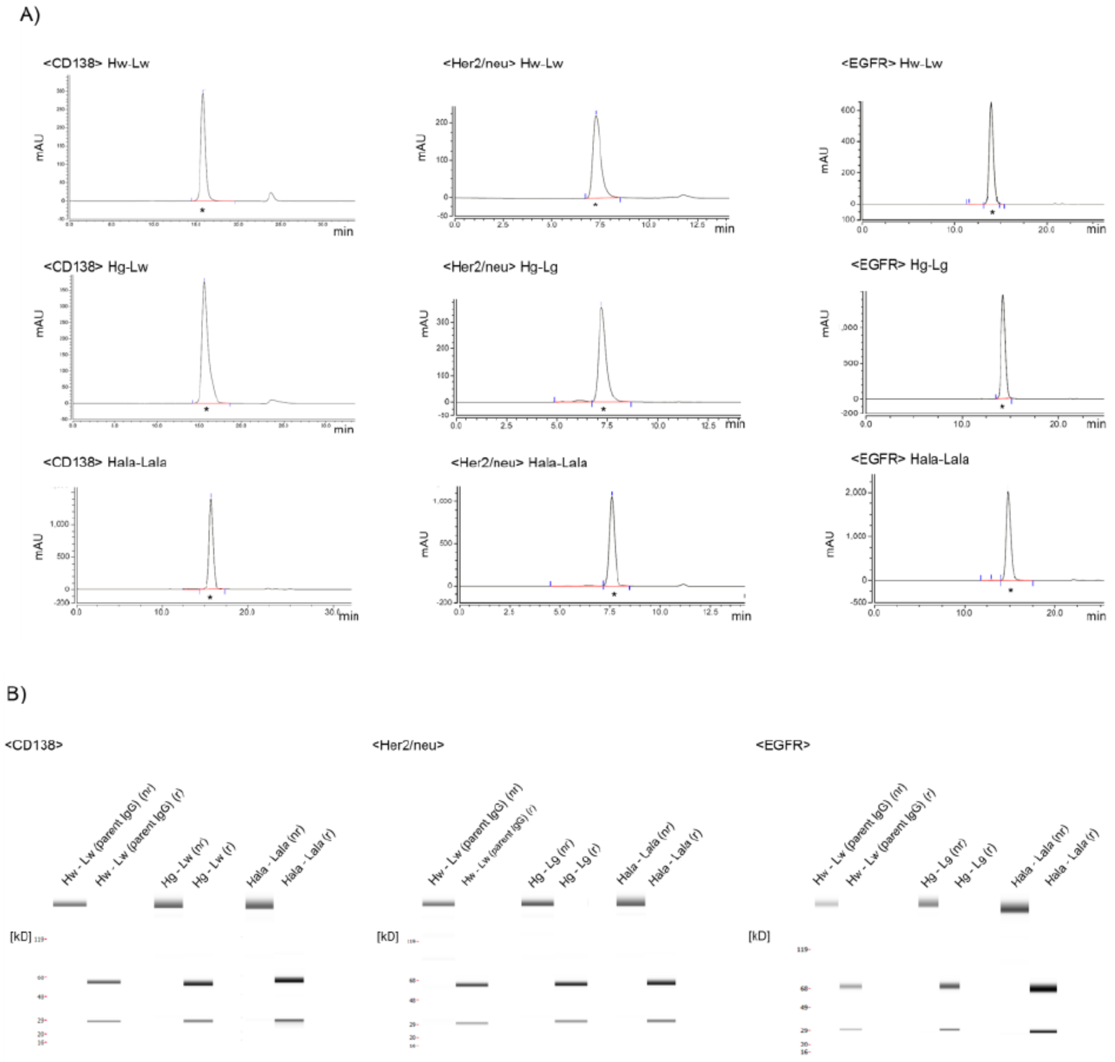

Figure S1. Analytical verification of B2G variants. Shown are representative variants of CD138, Her2/neu and EGFR binders. A) Analytical Size Exclusion Chromatography profiles (* indicates the collected fractions for mass analysis and experiments) and B) SDS page analysis of collected fractions (Caliper Life Science Lab Chip GxII, Perkin Elmer). Additionally, electrospray ionization mass spectra (acquired on a maXis Q-TOF (Bruker Daltonics, Bremen, Germany) equipped with a TriVersa NanoMate (Advion, Ithaca, NY)) were acquired (data not shown).

Table S1. Expression rates of antibodies and their B2G variants. Antibodies were expressed transiently in non-adherent HEK293F or HEK-Expi cells and purified using protein-A affinity and size-exclusion chromatography using an Äkta system (GE Healthcare) [38, 39]. None of the proteins behaved aberrantly and expression levels of affinity variants were comparable or higher as to their corresponding non-mutated WT IgG's. This indicates that the B2G tool-suggested mutations are well tolerated and that there is no influence of individual or combined B2G mutations on folding and antibody structure. There were no structural incompatibilities of H and L-variants upon combination within the B2G combination set and molecular mass was determined to be correct for each antibody.

Specificity	Expression System	Variant	Yield in mg/L
CD138	HEK293F	Hw-Lw (parent IgG)	47
		Hw- La	50
		Hw- Lg	49
		Ha – Lw	42
	Expi	Ha – La	216
		Ha – Lg	220
	HEK293F	Hb – Lw	50
		Hb – La	33
		Hb – Lg	48
		Hg - Lw	46
		Hg - La	56
		Hg - Lg	62
Her2	Expi	Hw-Lw (parent IgG)	47
		Hw-La	297
		Hw-Lb	175
		Hw-Lg	83
		Ha-Lw	392
		Hb-Lw	103
		Hg-Lw	170
		Hg-Lg	325
EGFR	HEK293F	Hw-Lw (parent IgG)	23
		Hw-La	50
		Hw-Lb	38
		Hw-Lg	72
		Ha-Lw	22
		Hb-Lw	23
		Hg-Lw	22
		Hg-Lg	46

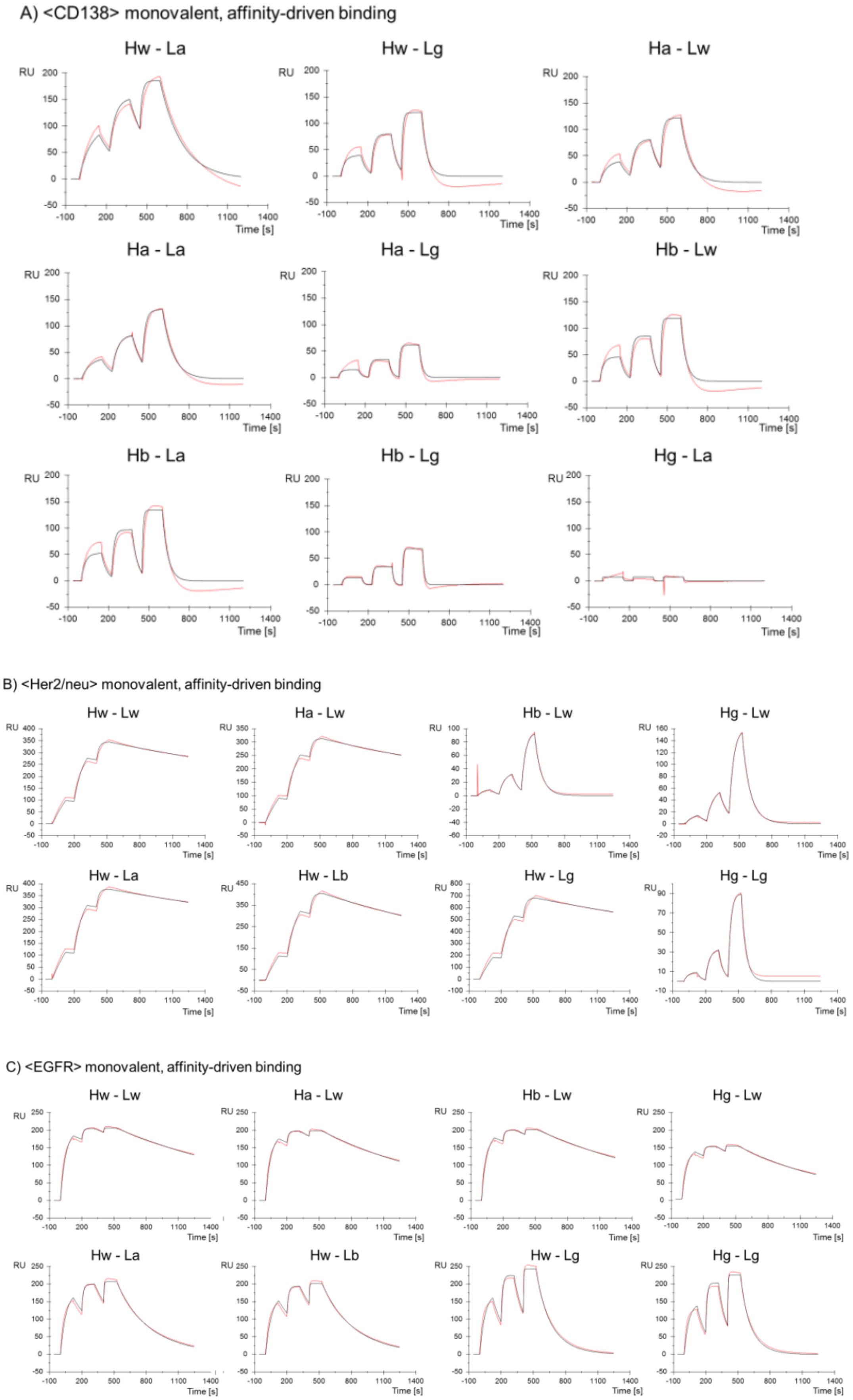

Figure S2. SPR Plots of all affinity measurements.

Table S2. Affinity modulation by B2G. Listed are On- (k_a) and Off-rate (k_d) as well as KD values of antibodies and corresponding B2G variants measured in a monovalent assay set up. Very small 'on' signals were observed for some CD138 binders (Hg-Lw, Hg-La and Hg-Lg; see Figure 2 and Figure S2) which may point towards weak interactions, which however cannot be called true binding events based on these 38 monovalent SPR analyses.

Specificity	VH-VL combination	k_a (1/Ms)	k_d (1/s)	KD (M)
CD138	Hw – Lw (parent IgG)	7.7E+04	6.2E-03	8.0E-08
	Hw – La	8.7E+04	6.1E-03	7.1E-08
	Hw – Lg	1.3E+04	2.5E-02	2.0E-07
	Ha – Lw	7.6E+04	1.5E-02	1.9E-07
	Ha – La	5.5E+04	1.3E-02	2.4E-07
	Ha – Lg	1.2E+05	4.5E-02	3.8E-07
	Hb – Lw	1.8E+05	2.7E-02	1.5E-07
	Hb – La	1.7E+05	2.5E-02	1.4E-07
	Hb – Lg	1.3E+05	7.9E-02	6.1E-07
	Hg – Lw	No valid result	No valid result	No valid result
	Hg – La	No valid result	No valid result	No valid result
	Hg – Lg	No valid result	No valid result	No valid result
Her2	Hw – Lw (parent IgG)	1.5E+05	2.8E-04	1,9E-09
	Hw – La	1.3E+05	2.5E-04	1,9E-09
	Hw – Lb	1.5E+05	4.1E-04	2,7E-09
	Hw – Lg	1.4E+05	2.6E-04	1,9E-09
	Ha – Lw	1.5E+05	3.1E-04	2,1E-09
	Hb – Lw	4.1E+04	1.6E-02	3,9E-07
	Hg – Lw	6.5E+04	2.2E-02	3,4E-07
	Hg – Lg	6.1E+04	2.4E-02	3,9E-07
EGFR	Hw – Lw (parent IgG)	1.07E+06	6.4E-04	6,0E-10
	Hw – La	9.97E+05	3.5E-03	3,5E-09
	Hw – Lb	8.90E+05	3.5E-03	3,9E-09
	Hw – Lg	1.00E+06	8.5E-03	8,5E-09
	Ha – Lw	1.06E+06	8.0E-04	7,3E-10
	Hb – Lw	1.06E+06	7.0E-04	6,6E-10
	Hg – Lw	1.13E+06	1.1E-03	9,7E-10
	Hg – Lg	1.08E+06	1.3E-02	1,2E-08

A) <CD138> bivalent, avidity-driven binding

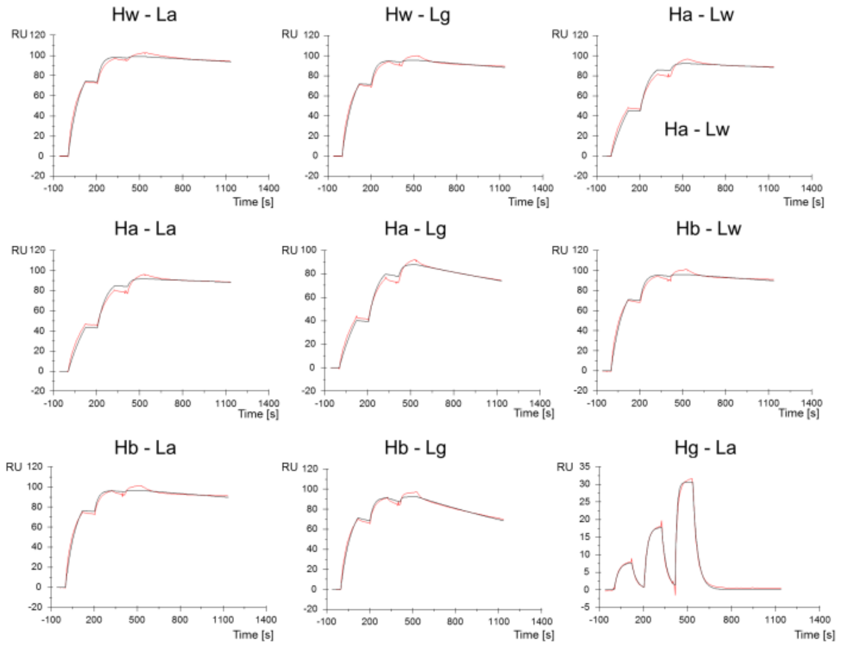

B) <Her2/neu> bivalent, avidity-driven binding

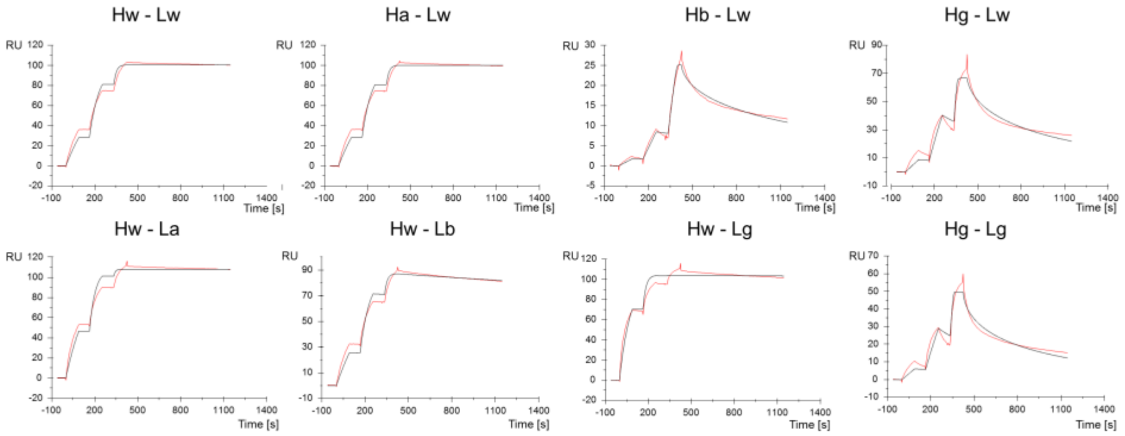

C) <EGFR> bivalent, avidity-driven binding

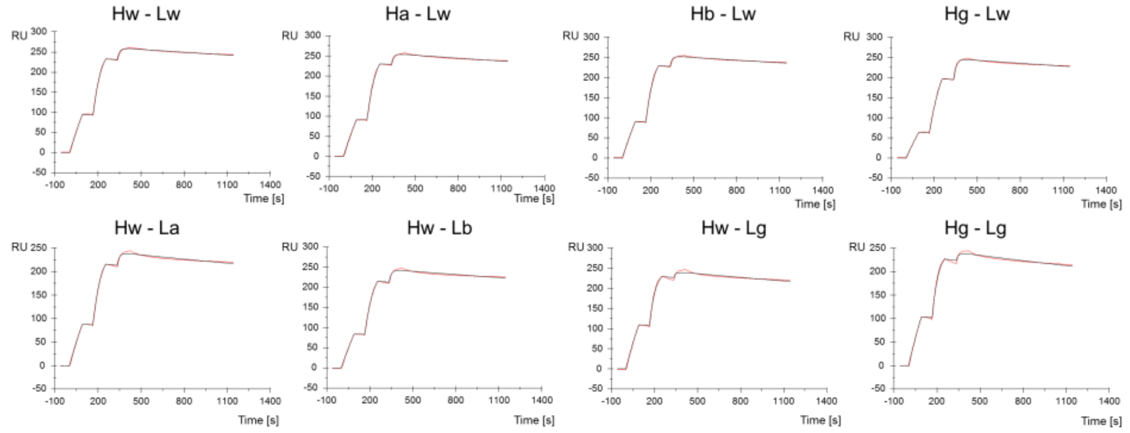

Figure S3. SPR Plots of all Avidity measurements.

Table S3. Avidity modulation by B2G. Listed are On- (k_a) and Off-rate (k_d) as well as KD values of antibodies and corresponding B2G variants measured in a bivalent assay set up. Very small 'on' signals were observed for CD138 binder Hg-Lg (see Figure 3B) which may point towards weak interactions, which however cannot be called true binding events based on these bivalent SPR analyses.

Specificity	VH-VL combination	k_a (1/Ms)	k_d (1/s)	KD (M)
CD138	Hw – Lw (parent IgG)	3.3E+05	7.8E-05	2,4E-10
	Hw – La	3.6E+05	8.7E-05	2,4E-10
	Hw – Lg	3.6E+05	1.3E-04	3,6E-10
	Ha – Lw	1.7E+05	7.1E-05	4,2E-10
	Ha – La	1.6E+05	6.4E-05	4,0E-10
	Ha – Lg	1.6E+05	2.9E-04	8,3E-10
	Hb – Lw	3.5E+05	1.1E-04	2,8E-10
	Hb – La	4.0E+05	1.2E-04	3,1E-10
	Hb – Lg	3.9E+05	5.0E-04	2,4E-09
	Hg – Lw	2.1E+05	5.1E-02	2,3E-07
	Hg – La	2.2E+05	3.8E-02	1,7E-07
	Hg – Lg	No valid result	No valid result	No valid result
Her2	Hw – Lw (parent IgG)	1.6E+05	7.0E-05	4,4E-10
	Hw – La	2.6E+05	7.1E-05	2,7E-10
	Hw – Lb	1.7E+05	1.7E-04	1,0E-09
	Hw – Lg	8.2E+05	1.3E-04	1,6E-10
	Ha – Lw	1.6E+05	7.1E-05	4,4E-10
	Hb – Lw	3.1E+04	2.1E-04	6,8E-09
	Hg – Lw	8.7E+04	1.5E-02	1,7E-07
	Hg – Lg	8.3E+04	1.5E-02	1,8E-07
EGFR	Hw – Lw (parent IgG)	1.5E+5	9.4E-05	6,3E-10
	Hw – La	1.6E+5	1.3E-04	8,1E-10
	Hw – Lb	1.5E+5	1.2E-04	8,0E-10
	Hw – Lg	1.4E+5	1.4E-04	1,0E-10
	Ha – Lw	1.5E+5	1.0E-04	6,7E-11
	Hb – Lw	4.1E+4	9.3E-05	2,3E-10
	Hg – Lw	6.5E+4	1.1E-04	1,7E-09
	Hg – Lg	6.1E+4	1.7E-04	2,8E-08

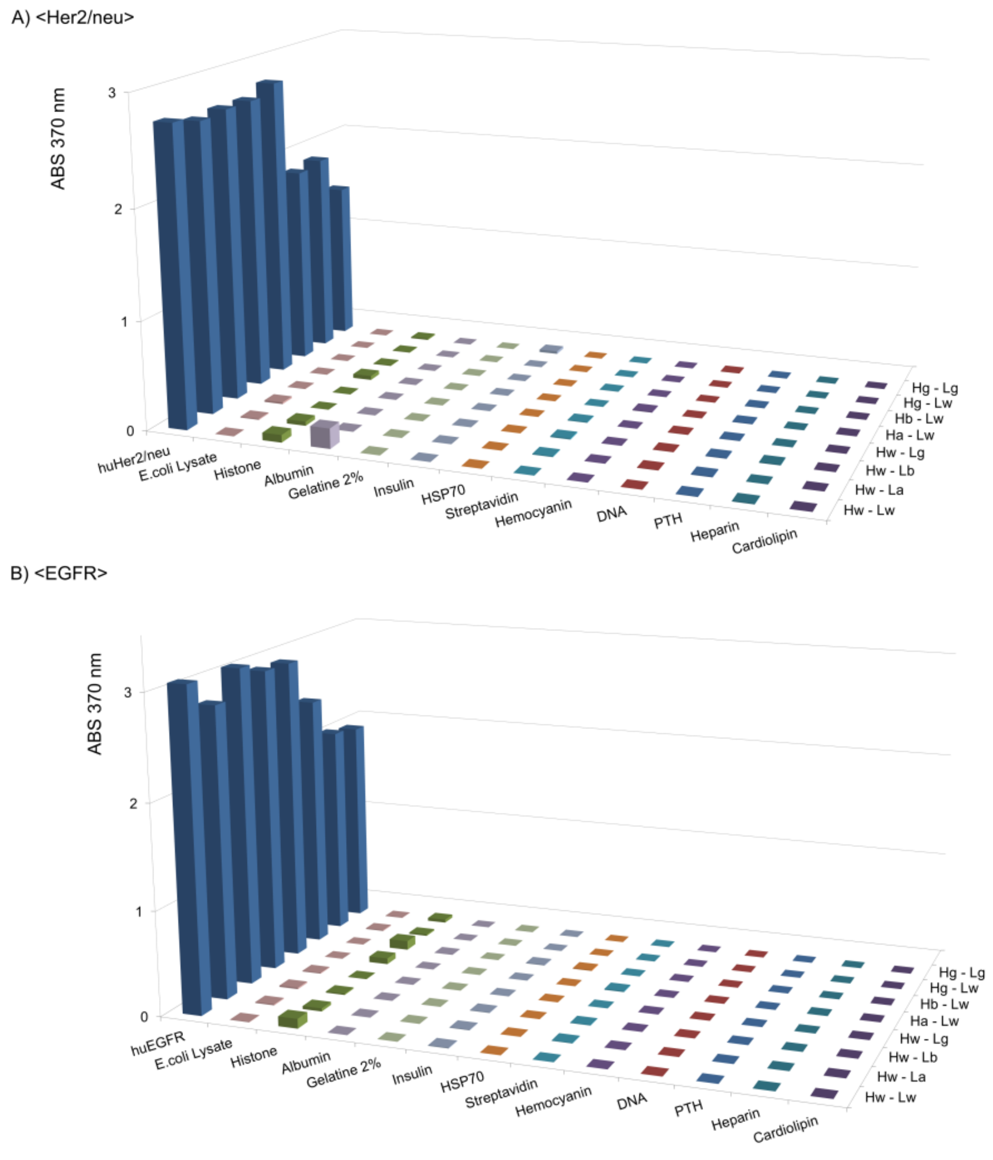

Figure S4. Polyreactivity assays of <Her2/neu> and <EGFR> B2G variants.

Table S4. Expression yields of antibody derivatives that harbor alanine at B2G-defined CDR positions. Antibodies were expressed transiently in non-adherent HEK293F or HEK-Expi cells and purified using protein-A affinity and size-exclusion chromatography using an Äkta system (GE Healthcare) 38, 39. To verify antibody identity electrospray ionization mass spectra were acquired on a maXis Q-TOF (Bruker Daltonics, Bremen, Germany) equipped with a TriVersa NanoMate (Advion, Ithaca, NY). Expression yields were comparable as to their corresponding B2G variants and non-mutated WT IgG's.

Specificity	Expression System	Variant	Yield in mg/L
CD138	HEK293F	Hala - Lala	114
Her2/neu	Expi	Hala - Lala	909
EGFR	HEK293F	Hala - Lala	152

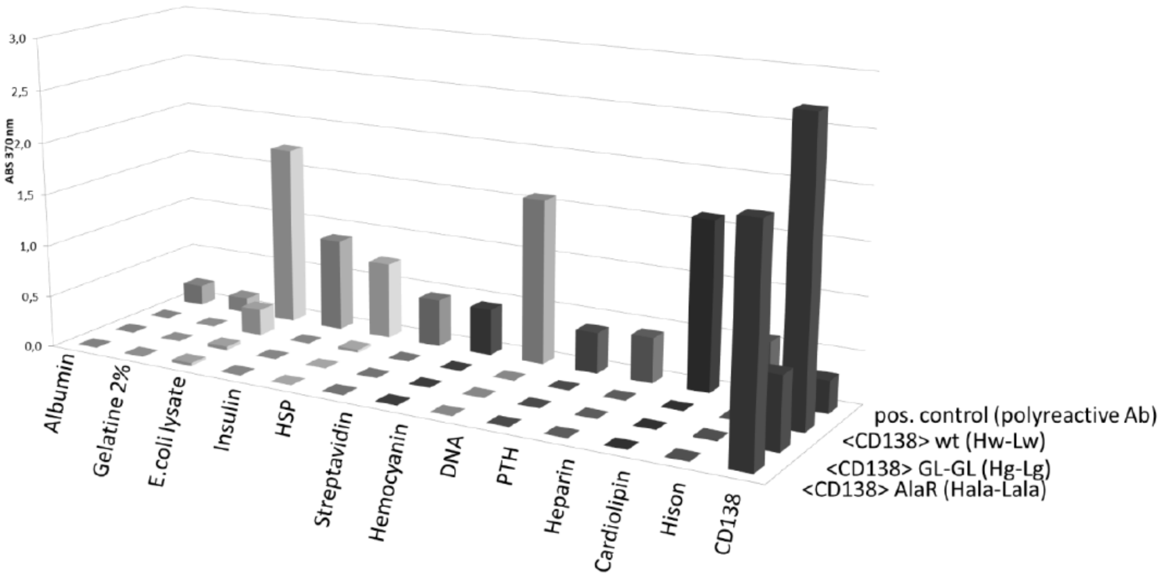

Figure S5. Polyreactivity assays of <CD138> variants.

Data 1. Differentiation of CDR residues by addressing probability of antigen interaction.

CDR residues were analyzed for antigen contact propensity, their positions identified by the WolfGuy numbering as previously described (* Bujotzek, A., Dunbar, J., Lipsmeier, F., Schäfer, W., Antes, I., Deane, C. M., & Georges, G. 2015: Prediction of VH–VL domain orientation for antibody variable domain modeling. Proteins: Structure, Function, and Bioinformatics, 83(4), 681-695). Solvent accessibility changes and chemical antigen interactions reflect antigen contacts and are defined as not detectable (-), detectable only in a very small number of antibodies (+/-) and detectable with increasing frequencies (+, ++, +++). CDR residues for which there were less than 50 non-redundant antibody-antigen complex structures available were omitted (blank fields). All X-ray structures considered had a resolution of 3.0 Å or higher.

Antigen contact propensity categories	-	+/-	+	++	+++
Average amino acid sidechain solvent accessibility change [%]	0	]0, 5.0]	]5.0, 10.0]	]10.0, 20.0]	> 20.0
Average number of chemical interactions with antigen	0	]0, 0.2]	]0.2, 0.4]	]0.4, 0.8]	> 0.8

		Average amino acid sidechain solvent accessibility change		Average number of chemical interactions with antigen	
WolfGuy* Index	Region	Protein Ag	Peptide Ag	Protein Ag	Peptide Ag
151	CDR-H1	+/-	-	-	-
152		+/-	+/-	+/-	+/-
153		+	+/-	+/-	+/-
154		+/-	+/-	+/-	-
155		+	+/-	+/-	+/-
156		+++	+	+	+/-
196		+	+	+	+
197		++	+++	++	+++
198		-	-	-	-
199		+/-	+/-	+/-	+/-
251	CDR-H2	+	++	++	++
252		+/-	-	-	-
253		++	++	+++	+++

254		++	++	+	+
255		+++	+	++	+/-
288		+++	+	++	+
289		+	+/-	+/-	+/-
290		+++	++	++	++
291		+/-	+/-	+/-	-
292		++	++	++	++
293		+/-	+/-	-	-
294		+/-	+/-	-	-
295		+	+/-	+/-	+/-
296		+/-	+/-	+/-	-
297		-	-	-	-
298		+/-	+/-	+/-	-
299		+/-	-	-	-
351	CDR-H3	+	++	+	++
352		++	++	++	+
353		+++	+++	++	++
354		+++	++	+++	++
355		+++	++	+++	+/-
356		+++	+	++	+/-
357		+++		+++	
390		+++		+++	
391		+++		+++	
392		+++	++	++	++
393		+++	+++	++	++
394		++	+	++	+
395		++	++	+++	++
396		+/-	+/-	+/-	+/-
397		+/-	+/-	+/-	+/-
398		+/-	+/-	+/-	+/-
399		+/-	+/-	+/-	+/-
551	CDR-L1	+/-	-	+/-	-
552		-	-	-	-
553		+/-	-	-	-
554		+/-		-	

556		+/-	+/-	+/-	+/-
561		+	+/-	+/-	+/-
562		+/-	+/-	+/-	+/-
563		+/-	+/-	+/-	+/-
571		+++	+	+	+/-
572		++		+	
581		+++	+++	++	+++
582		+++	++	++	+
583			++		+/-
594		++	+/-	+/-	-
595		++	+	++	+
596		++	+/-	+	+/-
597		+++	++	+++	+++
598		-	-	-	-
599		+/-	+/-	+/-	+
651	CDR-L2	++	+	++	+
694		+/-	+/-	+/-	+/-
695		+/-	+/-	+/-	-
696		++	+/-	+	+/-
697		+/-	-	+/-	-
698		+/-	+/-	+/-	+/-
699		+	+/-	+/-	-
751	CDR-L3	+/-	+/-	+/-	+/-
752		+/-	+/-	-	-
753		+	++	+	++
754		++	+	++	+
755		++	++	+	+
756		++		++	
795		+		+/-	
796		++	++	++	++
797		+/-	+/-	+/-	+/-
798		+	+	+	++
799		+/-	+/-	+/-	-
